# Targeting sympathetic glia for treating cardiovascular diseases

**Published:** 2017-08-07

**Authors:** Alison Xiaoqiao Xie, Angelo I. Chaia, Ken D. McCarthy

**Affiliations:** 1 Department of Pharmacology, UNC-Chapel Hill, Chapel Hill, NC, 27599-7365 USA; 2 Department of Chemistry, UNC-Chapel Hill, Chapel Hill, NC, 27599-3290 USA

**Keywords:** Gq-DREADD, pharmacogenetics, sympathetic ganglia, satellite glial cells, cardiovascular diseases, AAV

## Abstract

Gq G protein-coupled receptor (Gq-GPCR) signaling in glial fibrillary acidic protein-expressing (GFAP^+^) glia is essential for neuron-glia interaction in the Central Nervous System (CNS). However, the exploration of the roles of Gq-GPCR signaling in peripheral GFAP^+^ glia has just begun. Our recent study showed that GFAP^+^ glia in the sympathetic ganglia, namely satellite glial cells (SGCs), positively modulate sympathetic-regulated cardiac functions following their Gq-GPCR activation. In this research highlight, we discuss the significance of satellite glial modulation of sympathetic nerve activity (SNA) in both physiology and in diseases. We also present a new experimental strategy for manipulating satellite glial signaling in the sympathetic ganglia using adeno-associated virus (AAV). The success of targeted viral transduction in ganglionic SGCs suggest a strong therapeutic potential of targeting sympathetic glia for the treatment of cardiovascular diseases (CVDs).

## Background

Chronic sympathoactivation, diagnosed via increased sympathetic nerve activity (SNA)^[1]^, is correlated with hypertension in humans^[2, 3]^ as well as in experimental models of hypertension^[3]^. Elevated SNA, particularly to the heart and kidneys, leads to neurogenic hypertension^[3]^, which then contributes to multiple high-mortality diseases^[1, 3]^. What deters us from developing novel and effective treatments for neurogenic cardiovascular diseases (CVDs) is the significant gap in our understanding of SNA and its complex regulation *in vivo*.

**Figure 1:**
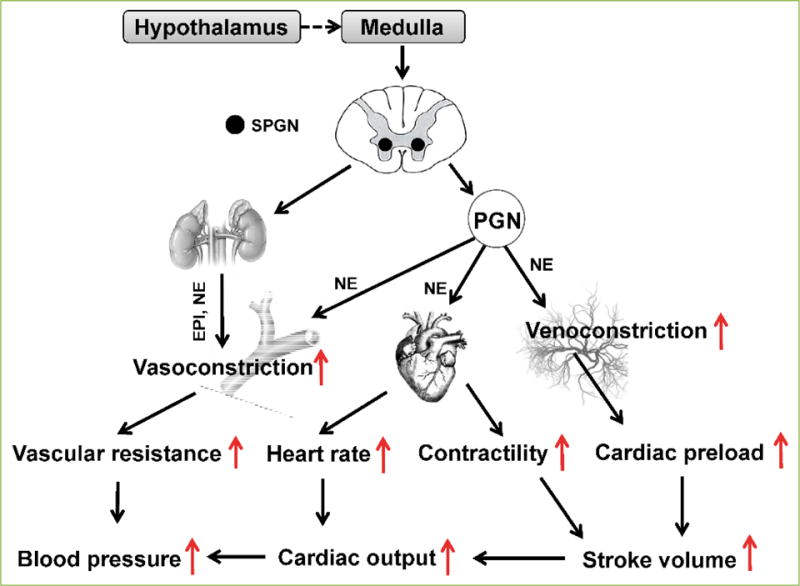
Cardiovascular adjustments with sympathetic activation Increased sympathetic activity from the medulla constricts the carotid and splanchnic arteries as well as venous vessels, increasing vessel distension and resistance. Increased sympathetic drive also increases heart rate and contractility, which, together, increase cardiac output. Increased cardiac output and vascular resistance lead to increased blood pressure. Venoconstriction also contributes to increased cardiac preload. SPGN: spinal pre-ganglionic neurons; PGN: post-ganglionic neurons; EPI: epinephrine; NE: norepinephrine

As a key controller of the cardiovascular system, sympathetic nerves are tonically active in a synchronized and rhythmic fashion^[1]^. Brain-originating rhythmical SNA is amplified via sympathetic preganglionic neurons (SPGN) in the spinal cord and postganglionic neurons (PGN) in sympathetic ganglia (Fig. 1). In the CNS, GFAP^+^ astrocytes reside in close proximity to active synapses^[4]^, and regulate neuronal activity^[5]^ and signal processing^[6]^ in an activity-dependent and circuit-specific manner^[7]^. Recent studies on enteric glia revealed novel mechanisms of GFAP^+^ glial regulation of gastrointestinal functions^[8, 9]^, suggesting powerful neuromodulatory potential of GFAP^+^ glia in the peripheral nervous system.

We began by testing the role of GFAP^+^ glia in the SNS *in vivo*. More specifically, we asked if the activation of Gq-GPCR signaling in sympathetic GFAP^+^ glia modulates sympathetic regulated physiology in awake and free-moving animals. Glial Gq-GPCR signaling is essential to neuron-glia interaction in the CNS^[10]^. The challenge to studying the role of Gq-GPCR signaling in GFAP^+^ glia is that neurons and GFAP^+^ glia express overlapping GPCRs^[11]^. Traditional pharmacological stimulation leads to Gq-GPCR activation in both neurons and GFAP^+^ glia, causing difficulties in dissecting the contribution of GPCR activation specifically in GFAP^+^ glia. More recent optogenetic methods paired with targeted viral delivery can selectively elevate intracellular calcium in GFAP^+^ glia, mimicking one of the downstream signaling effects following Gq-GPCR activation in these cells. However, optogenetic manipulation in GFAP^+^ glia fails to activate the extensive network of Gq-GPCR signaling pathway in GFAP^+^ glia^[11]^. Moreover, the activation of optogenetic channels on GFAP^+^ glia leads to strong depolarization and acidification^[12]^, which are not present in physiological glial Gq-GPCR activation. Therefore, we chose to use a pharmacogenetic approach in our studies to activate Gq-GPCR signaling in GFAP^+^ glia, by expressing engineered Gq-GPCRs only in GFAP^+^ glia but not neurons and other glial cells.

## A pharmacogenetic model for studying Gq-GPCR activation in GFAP^+^ glia in vivo

In order to selectively activate Gq-GPCR signaling pathway in GFAP^+^ glia without activating other cells types, we took advantage of the newly developed *Designer Receptors Exclusively Activated by Designer Drugs* (DREADDs)^[13, 14]^. DREADDs are engineered by introducing point-mutations to endogenous muscarinic receptors (mAChR)^[15]^. DREADDs can only be activated by the otherwise bio-inert small molecule clozapine-N-oxide (CNO), and such activation can be blocked by mAChR antagonists^[15]^. We generated GFAP-hM3Dq transgenic mice, in which the Gq-coupled DREADD, hM3Dq^[15]^, is exclusively expressed in GFAP^+^ glia in the CNS and the PNS^[13]^. hM3Dq expression was largely restricted in the nervous system, and no hM3Dq expression was detected on the target organs, including the heart and blood vessels^[13–15]^. CNO can cross the blood brain barrier (BBB), making the GFAP-hM3Dq transgenic mice a unique model for assessing the role of Gq-GPCR activation in GFAP+ glia *in vivo*^[16, 17]^.

Upon administration, CNO exclusively activates hM3Dq^[18]^ in GFAP^+^ glia in the nervous system. hM3Dq does not exhibit intrinsic activity in the absence of CNO^[19]^; thus, there are no baseline differences in physiology or behavior between GFAP-hM3Dq mice and wild-type littermate controls^[13]^. In contrast, a single Intraperitoneal injection (i. p.) of CNO leads to significant increases in both heart rate and left ventricle contraction in GFAP-hM3Dq mice^[14, 15]^. Using the GFAP-hM3Dq transgenic model, we provided clear pharmacological evidence supporting that peripheral GFAP^+^ glia, specifically satellite glial cells (SGCs) in the sympathetic ganglia, positively modulate sympathetic released norepinephrine (NE) onto the heart, and in turn significantly increases cardiac contractility^[14]^.

## What are SGCs and what do they (presumably) do?

Sympathetic ganglia consist of PGNs, axonal terminals from spinal SPGN, small intensely fluorescent cells (SIF), and GFAP^+^ SGCs. SGCs form peri-neuronal sheaths that tightly wrap around neuronal soma and axon-soma contacts in sympathetic ganglia^[20]^. SGCs effectively isolate individual PGNs^[20]^ and comprise an effective chemical barrier for the whole ganglion^[21]^, suggesting their potential role of governing PGN activity. Sympathetic SGCs also express machinery including inward rectifying potassium channels (Kir)^[22–24]^, Ca^2+^-activated potassium channels^[24]^, gap junctions^[24, 25]^, neurotransmitter transporters^[26–29]^, enzymes for neurotransmitter degradation and synthesis^[30]^, and metabotropic neurotransmitter receptors^[31, 32]^, further suggesting their important roles in modulating ganglionic neuronal activity and signaling processing. Recent studies in sensory ganglia revealed bi-directional purinergic signaling between ganglionic neurons and sensory SGCs^[33, 34]^, indicating the potential contribution of SGC activation in neurogenic chronic pain. However, in sympathetic ganglia, the roles of SGCs in regulating local neuronal activity and sympathetically-driven physiology had not been reported prior to our study^[35]^. Our findings suggest that Gq-GPCR signaling in SGCs directly increases PGN activity in sympathetic ganglia and subsequently enhanced sympathetic-regulated physiology. This is the first report on the function of ganglionic SGCs in sympathetic ganglia.

Our study also expanded the field of GFAP^+^ glia-neuron interaction from the CNS to the PNS. PNS GFAP^+^ glia consists of SGCs in all types of ganglia and non-myelinated schwann cells (NMSC; also called terminal schwann cells (TSC)) near the nerve endings in target organs (including muscles). However, the role of peripheral GFAP^+^ glia is largely overlooked. Our findings strongly argue that GFAP^+^ glia in the PNS can directly modulate the activity of local neural network, and exhibit profound influences on target organ functions following their Gq-GPCR activation. GFAP^+^ glia express many metabotropic neurotransmitter receptors that are GPCRs. The manipulation of Gq-GPCR signaling in peripheral GFAP^+^ glia may present a powerful tool to manipulate target organ function from the point of peripheral ganglia/nerve endings.

## Can we target SGC signaling in the sympathetic ganglia for treating CVDs?

Within the growing population of hypertensive patients (global prevalence projected to reach one billion by 2025^[36]^), 30% have drug-resistant hypertension^[37]^ and their disease progression can only be managed by clinical strategies to decrease SNA^[2]^. Clinical strategies of decreasing SNA includes central sympatholytics^[38]^, deep brain stimulation^[39]^, regional sympathectomy^[40]^, and chronic carotid sinus baroreceptor stimulation^[41]^. These procedures often involve surgeries and device implantation, and they are generally irreversible. The safety and efficacy of these procedures are still being established within the clinical community.

**Figure 2:**
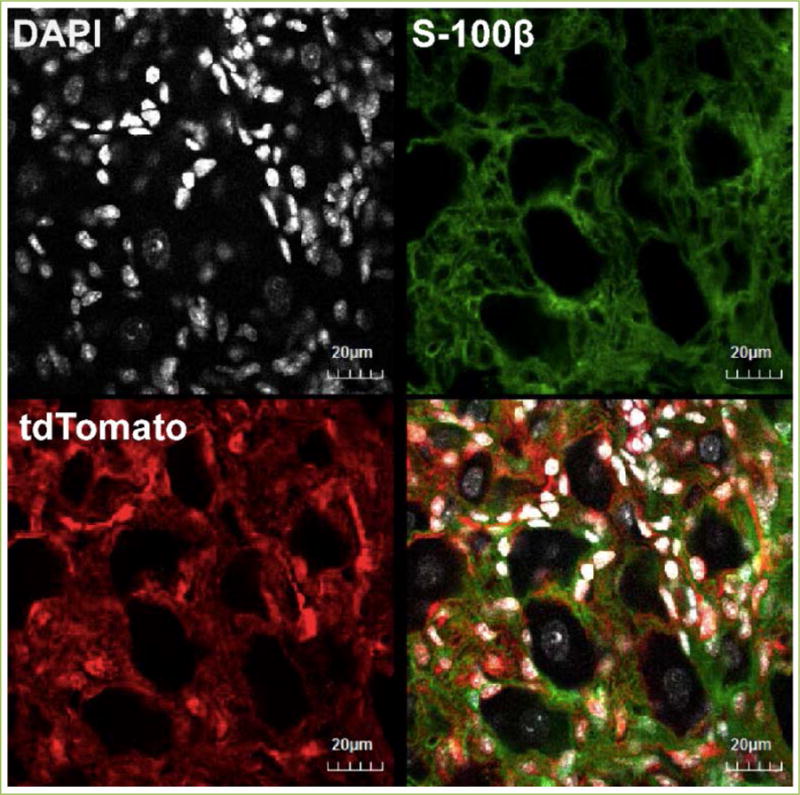
Tdtomato expression in SGCs, but not PGNs, in the superior cervical ganglia of Rosa26-Ai9 Cre reporter mice, 4 weeks after AAV8-GFAP-Cre injection Scale bar: 20 μm.

The causal link between hM3Dq activation in ganglionic satellite glia and the enhanced cardiac functions suggests strong therapeutic potential of selective manipulation of sympathetic SGC signaling in CVD treatment. In our study, we also found that chronic activation of satellite glial Gq-GPCR signaling led to significant decreases in blood pressure in female GFAP-hM3Dq mice, suggesting a strong link between sympathetic SGC Gq-GPCR signaling and blood pressure regulation^[14]^. However, can we manipulate Gq-GPCR signaling exclusively in sympathetic SGCs *in vivo*? Recently, we have optimized protocols for 1) targeted AAV viral injections into superior cervical ganglia and 2) AAV-mediated gene expression for high-efficiency and high tropism towards SGCs in superior cervical ganglia. The injection technique and the AAV viral vector enable selective manipulation of SGC signaling pathways *in vivo* (Fig. 2).

In brief, naïve C57BL/6 mice (in both sexes) were maintained under general anesthesia using isoflurane. For each mouse, a ventral, medial incision at the neck was made and tissue was separated until the esophagus of the mouse was visible. The muscles and glands were carefully pushed to the side to expose the superior cervical ganglia on both sides. For each ganglion, an injector apparatus was lowered into the ganglion using standard stereotaxic procedure. After puncturing the connective tissue around the ganglion, 500 nL of AAV vectors were infused into each superior cervical ganglion over 5 minutes at a rate of 0.1 μL per minute. The needle was kept in for another five minutes before removed. After both ganglia were injected, the tissues were moved back to their original place and the incision was closed with Vet Bond and Liquid Bandage. Lidocaine and ciprofloxacin were injected during the post-surgery recovery to manage pain and potential infections. The protocol for this procedure is approved and were conducted in accordance with Institutional Animal Care and Use Committee (IACUC) guidelines at University of North Carolina at Chapel Hill.

**Figure 3:**
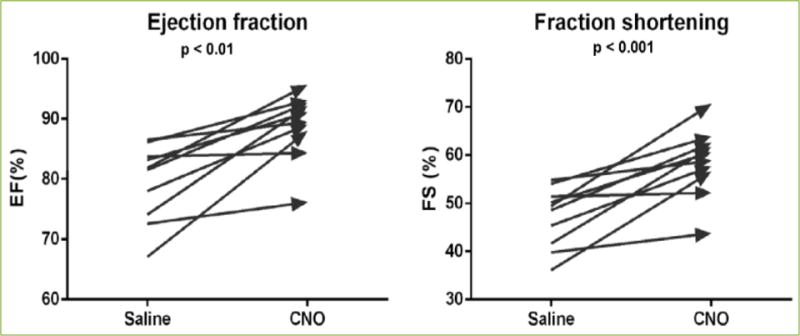
Ejection fraction (EF) and fraction shortening (FS) recorded from naïve mice injected with AAV8-GFAP-hM3Dq-tdTomato Both EF and FS significantly increased in response to a single i. p. injection of CNO. The increases are comparable to those in GFAP-hM3Dq mice after CNO administration.

We chose AAV8 serotype for its relatively higher tropism towards glial cells^[42]^ and low-probability of inducing innate immune responses^[43]^. Our preliminary data demonstrated that injecting AAV8-GFAP-Cre (1.0 × 10^13^ vg/μL) into the superior cervical ganglia of Rosa26-Ai9 Cre reporter mice^[44]^ led to tdTomato expression in the majority of SGCs, with no apparent neuronal transduction (Fig. 2). Furthermore, naïve mice injected with AAV8-GFAP-hM3Dq-tdTomato (0.5~1.5 × 10^13^ vg/μL) responded to i. p. CNO administration with increases in left ventricle ejection fraction and fraction shortening that were comparable to those observed in GFAP-hM3Dq mice (Fig. 3). These data strongly demonstrate our ability to use AAV-mediated viral approach for selective manipulation of SGC signaling.

Sensory SGCs have been targeted for gene therapies treating chronic pain^[45–47]^. Direct injections of adenoviral vectors into the rat trigeminal ganglia^[46]^ and rat dorsal root ganglia^[48]^ leads to a sustained expression of the delivered genes in SGCs. Adenoviral transduction of glutamic acid decarboxylase (GAD) into SGCs resulted in glial production of GABA and reduced pain behavior *in vivo*^[46]^, suggesting a strong potential for altering ganglionic output by manipulating SGC signaling in the sensory ganglia. Our preliminary data strongly argues that targeted AAV injection into sympathetic ganglia leads to sustained and stable expression of hM3Dq in sympathetic SGCs without any detectable expression in PGNs. Injections into stellate ganglia, the sympathetic ganglia that innervate heart in human, are performed routinely in patients to control sympathetic output^[49]^. Future pre-clinical research is required to determine the long-term effect of overexpressing engineered Gq-GPCRs in SGCs on cardiovascular physiology, as well as to assess the long-term effects of activating satellite glial Gq-GPCR signaling on cardiovascular functions.

## Abbreviations

AAV: adeno-associated virus
BBB: blood brain barrier
CNS: central nervous system
CVD: cardiovascular disease
DREADD: Designer Receptors Exclusively Activated by Designer Drugs
GAD: glutamic acid decarboxylase
GFAP: glial fibrillary acidic protein
GPCR: G-protein-coupled receptor
NE: norepinephrine
mAChR: muscarinic receptors
NMSC: non-myelinating Schwann cells
PNS: peripheral nervous system
PGN: postganglionic neuron
SGC: satellite glial cell
SIF: small intensely fluorescent cells
SNA: sympathetic nerve activity
SNS: sympathetic nervous system
SPGN: sympathetic preganglionic neurons
TSC: terminal schwann cells
